# PRIORITIZING FUTURE RESEARCH ON NONDRUG DEMENTIA CARE INTERVENTIONS

**DOI:** 10.1093/geroni/igac059.1987

**Published:** 2022-12-20

**Authors:** Eric Jutkowitz, Peter Shewmaker, Fernando Alarid-Escudero

**Affiliations:** Brown University, Providence, Rhode Island, United States; Brown University, Providence, Rhode Island, United States; Center for Research and Teaching in Economics (CIDE), Mexico City, Aguascalientes, Mexico

## Abstract

We previously used an evidence-based mathematical model to evaluate the cost-effectiveness of psychosocial interventions that reduce the risk of a nursing home admission for people with dementia from a healthcare payer perspective. We found the incremental cost-effectiveness of MIND, an in-home intervention for people with mild-moderate dementia, compared to usual care was $271,456 per quality-adjusted life-year (QALY). The incremental cost-effectiveness of NYU Caregiver Intervention (NYUCI), which is for people with moderate dementia, compared to usual care was $3,964/QALY. Here we quantify the uncertainty around our cost-effectiveness estimates. First, we calculated the expected value of partial perfect information (EVPPI), which is the value of eliminating uncertainty around the treatment effect (i.e., risk of entering a nursing home) of MIND and NYUCI, and represents the maximum willingness-to-pay for a study to inform these estimates. Given a willingness-to-pay of $110,000/QALY, population EVPPI for MIND and the NYUCI were $81,000,000 and $395,000,000, respectively. Second, we calculated the expected value of sample information (EVSI), the expected net benefit of sampling (ENBS) and the optimal sample size (OSS). EVSI is the amount of uncertainty reduced from a pragmatic trial evaluating the risk of entering a nursing home for people in the intervention compared to usual care. ENBS is the return of a pragmatic trial with a fixed ($1,050,000) and per person ($2,000) cost to conduct the study. The OSS is the sample size that maximizes ENBS and was 3,571 for MIND and 5,357 for NYUCI. There is value in conducting pragmatic trials on MIND and NYUCI.

